# Ketamine-Induced Alteration of Working Memory Utility during Oculomotor Foraging Task in Monkeys

**DOI:** 10.1523/ENEURO.0403-20.2021

**Published:** 2021-04-05

**Authors:** Ryo Sawagashira, Masaki Tanaka

**Affiliations:** 1 Department of Physiology, Hokkaido University School of Medicine, Sapporo 060-8638, Japan; 2 Department of Psychiatry, Hokkaido University School of Medicine, Sapporo 060-8638, Japan

**Keywords:** central executive, exploratory behavior, NMDA receptor antagonist, nonhuman primate, recursive choice, visual search

## Abstract

Impairments of working memory (WM) are commonly observed in a variety of neurodegenerative disorders but they are difficult to quantitatively assess in clinical cases. Recent studies in experimental animals have used low-dose ketamine (an NMDA receptor antagonist) to disrupt WM, partly mimicking the pathophysiology of schizophrenia. Here, we developed a novel behavioral paradigm to assess multiple components of WM and applied it to monkeys with and without ketamine administration. In an oculomotor foraging task, the animals were presented with 15 identical objects on the screen. One of the objects was associated with a liquid reward, and monkeys were trained to search for the target by generating sequential saccades under a time constraint. We assumed that the occurrence of recursive movements to the same object might reflect WM dysfunction. We constructed a “foraging model” that incorporated (1) memory capacity, (2) memory decay, and (3) utility rate; this model was able to explain more than 92% of the variations in behavioral data obtained from three monkeys. Following systemic administration of low dosages of ketamine, the memory capacity and utility rate were dramatically reduced by 15% and 57%, respectively, while memory decay remained largely unchanged. These results suggested that the behavioral deficits during the blockade of NMDA receptors were mostly due to the decreased usage of short-term memory. Our oculomotor paradigm and foraging model appear to be useful for quantifying multiple components of WM and could be applicable to clinical cases in future studies.

## Significance Statement

Working memory (WM) is often difficult to quantitatively assess in clinical cases, although deficiencies in WM have been reported in a variety of disorders. Here, we developed a novel oculomotor foraging paradigm and devised a relevant model for accurately evaluating several parameters of WM during visual search. We applied it to monkeys with and without administration of low-dose ketamine, which has been used to produce animal models of schizophrenia. Subanesthetic doses of ketamine dramatically reduced the use of short-term memory and increased the rate of exploratory choice, whereas the changes in memory capacity and memory decay were only modest. Our behavioral paradigm and the proposed model will provide a better understanding of the pathophysiology of WM dysfunction.

## Introduction

Working memory (WM) is a critical cognitive function for goal-directed behaviors in everyday situations such as writing on the phone, cooking multiple dishes simultaneously, and exploring in messy rooms (Goldman-Rakic, 1995; Christophel et al., 2017). According to the multiple component model of WM (Baddeley and Hitch, 1974; Baddeley and Della Sala, 1996), it contains several functional modules for storing and manipulating temporary information. These functions are thought to be implemented in the prefrontal cortex (PFC; Fuster and Alexander, 1971; Mishkin and Manning, 1978; Funahashi et al., 1989; Miller et al., 1996; Courtney et al., 1997; Constantinidis et al., 2001), and their defects are found in many neurodegenerative disorders including schizophrenia (Elvevåg and Goldberg, 2000; Goldman-Rakic et al., 2004; van Os and Kapur, 2009; Lett et al., 2014). Currently, most rating scales of PFC functions evaluate the capacity of visual WM with a few exceptions that also evaluate the ability to update short-term memory (Shallice, 1982; Heaton et al., 1993; Della Sala et al., 1999). However, these clinical tests are usually time-consuming and their procedures are a bit complicated so that small children and severe patients often have a difficulty in performing them.

Multiple components of WM could be assessed with a much simpler visual search task. To achieve efficient search, one must need to remember the items previously examined (Gilchrist and Harvey, 2000; Peterson et al., 2001; Aks et al., 2002; Boot et al., 2004; Dickinson and Zelinsky, 2005; Beck et al., 2006; but see Horowitz and Wolfe, 1998; Woodman et al., 2001). Indeed, many psychophysical studies on visual search have reported a phenomenon called “inhibitory tagging” that reduces the allocation of attention to previously visited items (Klein, 1988; Müller and von Mühlenen, 2000; Takeda and Yagi, 2000; Snyder and Kingstone, 2007; Wang and Klein, 2010). The underlying neural mechanism of inhibitory tagging has also been examined using functional imaging (Lepsien and Pollmann, 2002; Mayer et al., 2004; Zhou and Chen, 2008) and in experimental animals (Mirpour et al., 2009, 2019), and evidence show that the priority map represented in the frontoparietal attention system dynamically alters during visual search (Itti and Koch, 2001; Bisley and Goldberg, 2010; Veale et al., 2017). Because the priority map is thought to be under strong top-down control, the visual WM system in the PFC is likely to provide spatial information that quickly modulates the priority map in the parietal cortex (Zelinsky and Bisley, 2015). In support with this, a recent study has shown that the occurrence of recursive behavior during visual search significantly correlates with the capacity of visual WM evaluated in the change detection task in individual subjects (Shen et al., 2014).

In this study, we developed an oculomotor foraging task to assess visual short-term memory that is inherently required for efficient search (Dickinson and Zelinsky, 2007). In this task, the subjects were presented with many identical objects and searched for a target by making sequential saccades under time constraints. As with the concentration (Pelmanism) game, an efficient search requires short-term memory to avoid revisiting the same items. We trained three macaque monkeys to perform the task and then quantitatively assessed their WM performance using a novel foraging model that referenced the previous models of visual search (Horowitz, 2006; Mirpour et al., 2009). Furthermore, we systemically administered low doses of ketamine (N-Methyl-D-Aspartate receptor antagonist) to the animals to mimic the pathophysiology of schizophrenia (Condy et al., 2005; Skoblenick and Everling, 2012; Blackman et al., 2013; Gil-da-Costa et al., 2013; Wang et al., 2013) and evaluated the changes in the model parameters.

## Materials and Methods

### Animal preparation and surgery

Three adult macaque monkeys (two males and one female, 5–9 kg) were used. All experimental protocols were approved in advance by the Hokkaido University Animal Care and Use Committee. The animals were sterilely implanted with head holders and an eye coil in separate surgical procedures under general isoflurane anesthesia. Analgesics (pentazocine and ketoprofen) were administered during and for a few days after each procedure. The animals were trained on oculomotor tasks after they were fully recovered from the surgeries. During the training and experimental sessions, animals sat in a primate chair with their heads restrained in a dark and sound-attenuated booth. Horizontal and vertical eye positions were recorded using the search coil technique.

### Visual stimuli and behavioral tasks

**Figure 1.**
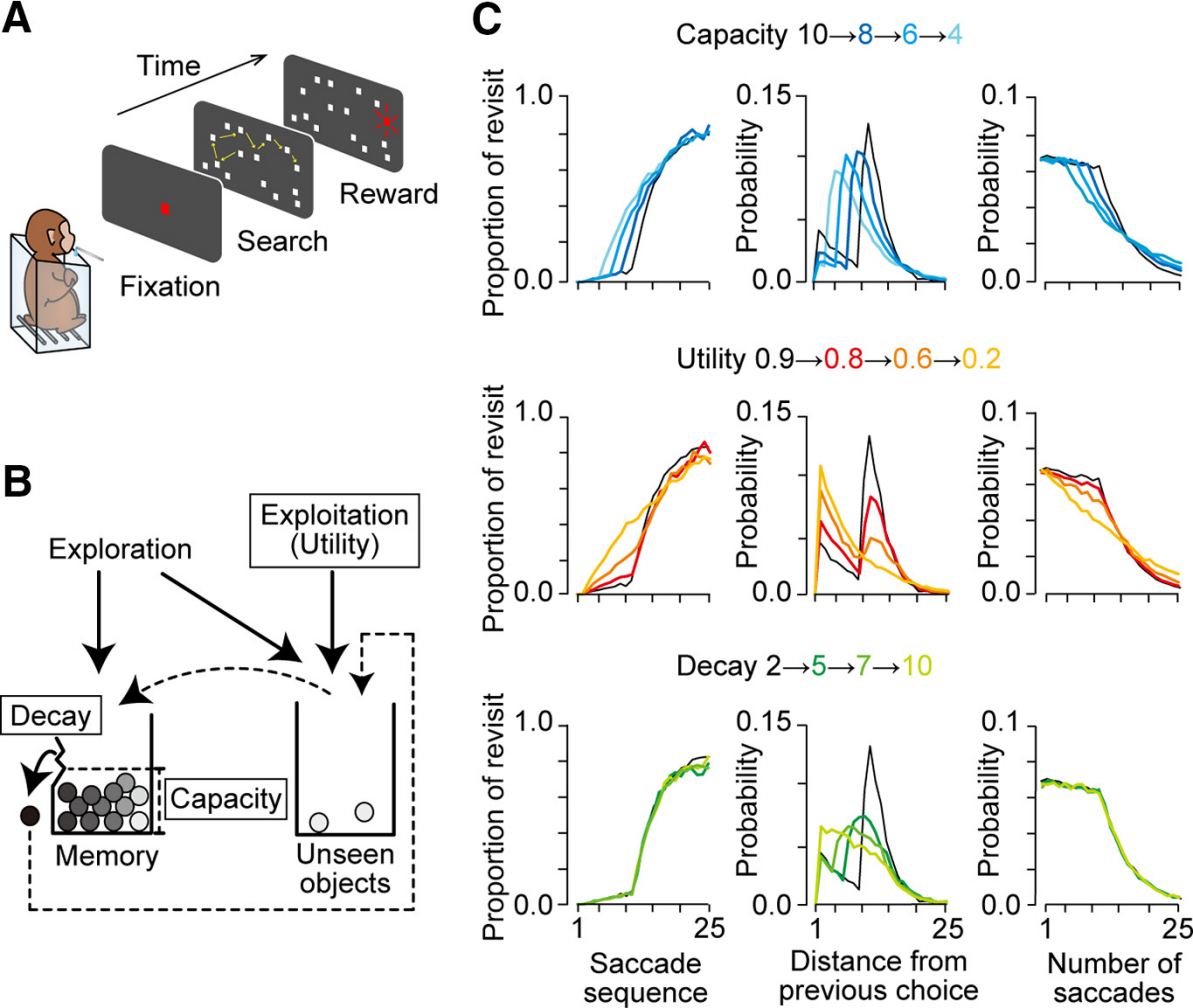
Behavioral task and the foraging model. ***A***, The oculomotor foraging task. Monkeys were presented with 15 identical objects (white squares) on the screen after the initial fixation period. One of the objects was associated with a liquid reward, and animals obtained a reward when they looked at the target for 100 ms within 6 s. As monkeys found the target, it turns red and the trial was terminated. ***B***, A schematic of the foraging model. Each circle represents a single object. During the visual search, the visited item was sequentially registered to the short-term memory and moved from the right box (unseen objects) to the left box (memory). When the number of items reached the memory capacity (12 items in this example), one item in the left box dropped off and moved back to the right box. The order of memory loss was defined by the memory decay and smaller number indicated better retention of short-term memory. In the exploration mode, the item was randomly chosen from 14 objects (except for the currently fixated object) with no reference to WM. Thus, the model was defined by three parameters: memory capacity, memory decay, and utility rate. ***C***, Model prediction derived from Monte Carlo simulations. Each column plots the relationship between saccade sequence and proportion of revisiting behavior (left), relative frequency of revisiting behavior as a function of the number of intervening saccades (distance) from the previous choice (middle), and relative frequency of saccade number in each trial. Each row compares the predictions for different memory capacities (top), utility rates (middle row), and memory decays (bottom). In all panels, the black traces indicate the data obtained from the model with memory capacity of 10, utility rate of 0.9, and memory decay of 2.

The experiments were controlled by a real-time data acquisition system (TEMPO, Reflective Computing). Visual stimuli were presented on a 27-inch liquid crystal display (XL2720Z, BenQ; refresh rate, 144 Hz) located 40 cm from the eyes (visual angle, 77° × 62°). In the oculomotor foraging task (Fig. 1*A*), each trial began with the appearance of a fixation point (FP; 0.6° red square) located either at the center of the display or at one of four peripheral positions (10° from the center and in the four 45° oblique directions). Fixation was detected when the eye position entered within 3° of the FP. After a random fixation period of 500–1000 ms, the FP disappeared and 15 white squares (0.6°, >4° apart from each other) were simultaneously presented for 6 s. The locations of the stimuli were randomly chosen for each trial from 76 possible sites covering the 25° × 40° area (7 × 11 grid of 4°, excluding the initial fixation location). One of the objects was associated with a liquid reward. Monkeys were trained to make sequential saccades until they received a reward within the 6-s time limit. During the experiments, saccades were detected when eye position remained within 2° of each stimulus for >100 ms. Once the monkeys looked at the target associated with a reward, the target turned red, and a liquid reward was delivered after a 200-ms delay period. After the reward was delivered, the trial was terminated with a brief high-frequency sound (1200 Hz, 100 ms). If monkeys failed to find the target by the end of the time limit, the trial was aborted with a pair of beep sounds (50 Hz, 100 and 500 ms). The intertrial interval was always 800 ms.

### Experimental procedures

**Figure 2.**
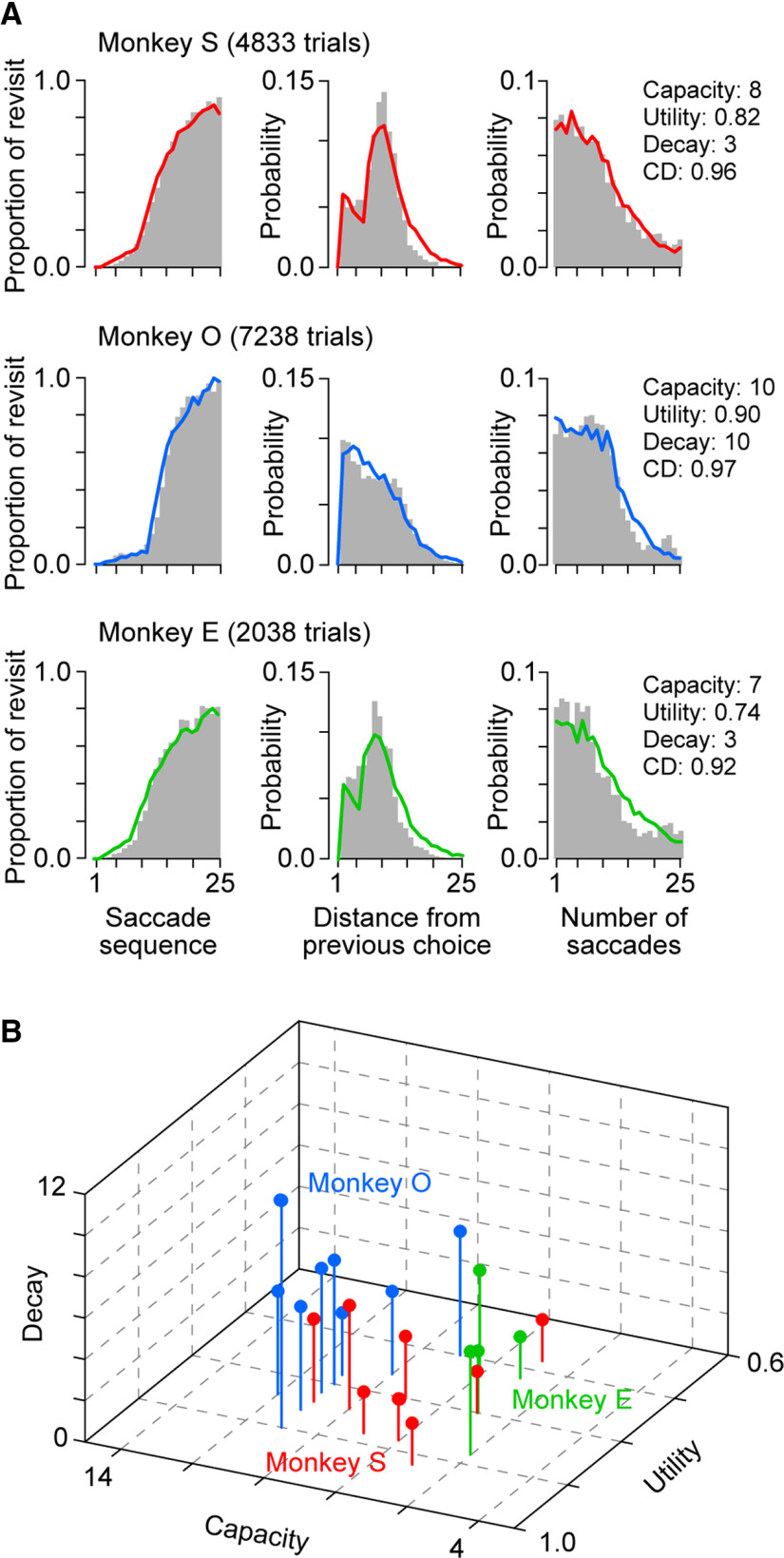
Behavioral data and model fitting. ***A***, Actual behavioral data (gray bars) were compared with the best-fit distributions obtained from the foraging model (colored lines). For each animal, data from multiple sessions were combined (nine, nine, and four sessions for monkeys S, O, and E, respectively). The CDs for the fit of each model were 0.96, 0.97, and 0.92 for monkeys S, O, and E, respectively. Note that when we evaluated the goodness-of-fit, each of the three distributions with 25 bins was normalized so that the area under the curve equaled unity. Three optimal parameters for each animal are reported in the right panel. ***B***, Optimal parameters in individual sessions. Note that the data from each monkey separated by color are clustered.

**Figure 3.**
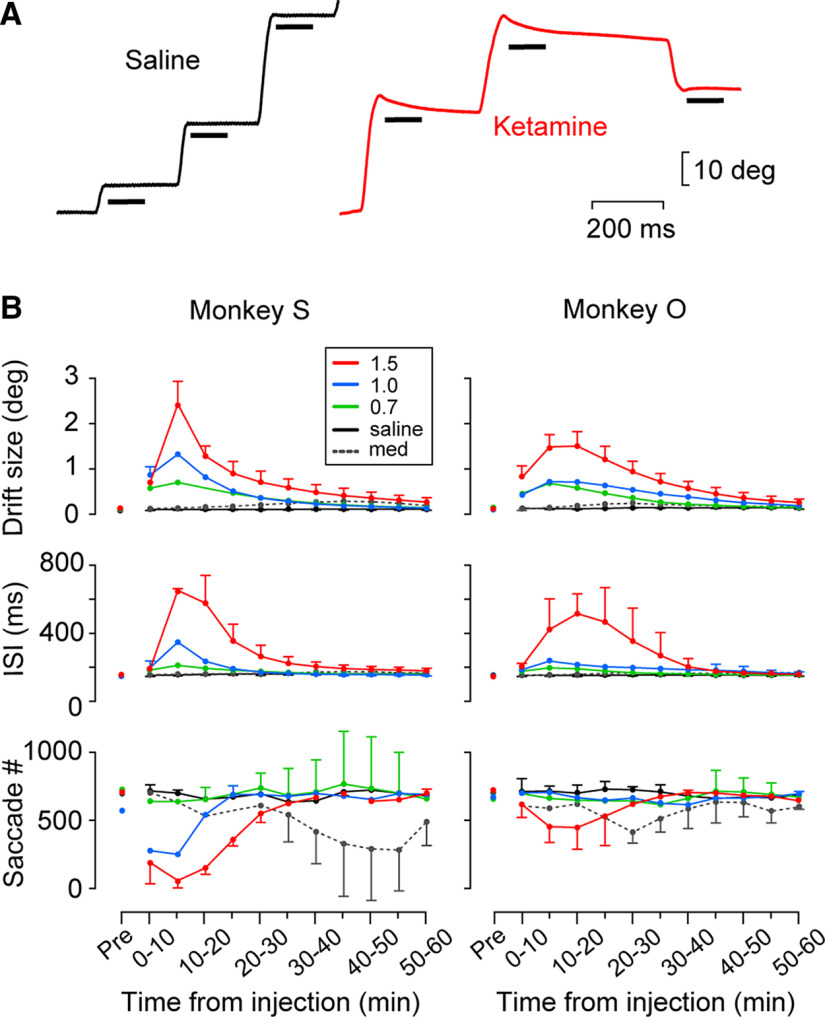
Effects of ketamine administration on oculomotor parameters. ***A***, Eye position traces from single trials after saline (black) or ketamine (1.5 mg/kg, red) injection in monkey S. The horizontal bar indicates the 100-ms interval for measuring postsaccadic drift. ***B***, Time course of eye movement parameters (drift size, ISI, and saccade number in every 10 min) following intramuscular injection of saline or drugs (0.7, 1.0, and 1.5 mg/kg ketamine and 0.01 mg/kg medetomidine). Data for the same experimental condition (three sessions) are connected with lines. Different colors indicate different drug conditions. The error bar indicates 1 SD and is only displayed for the maximal or minimal values of each time window.

**Figure 4.**
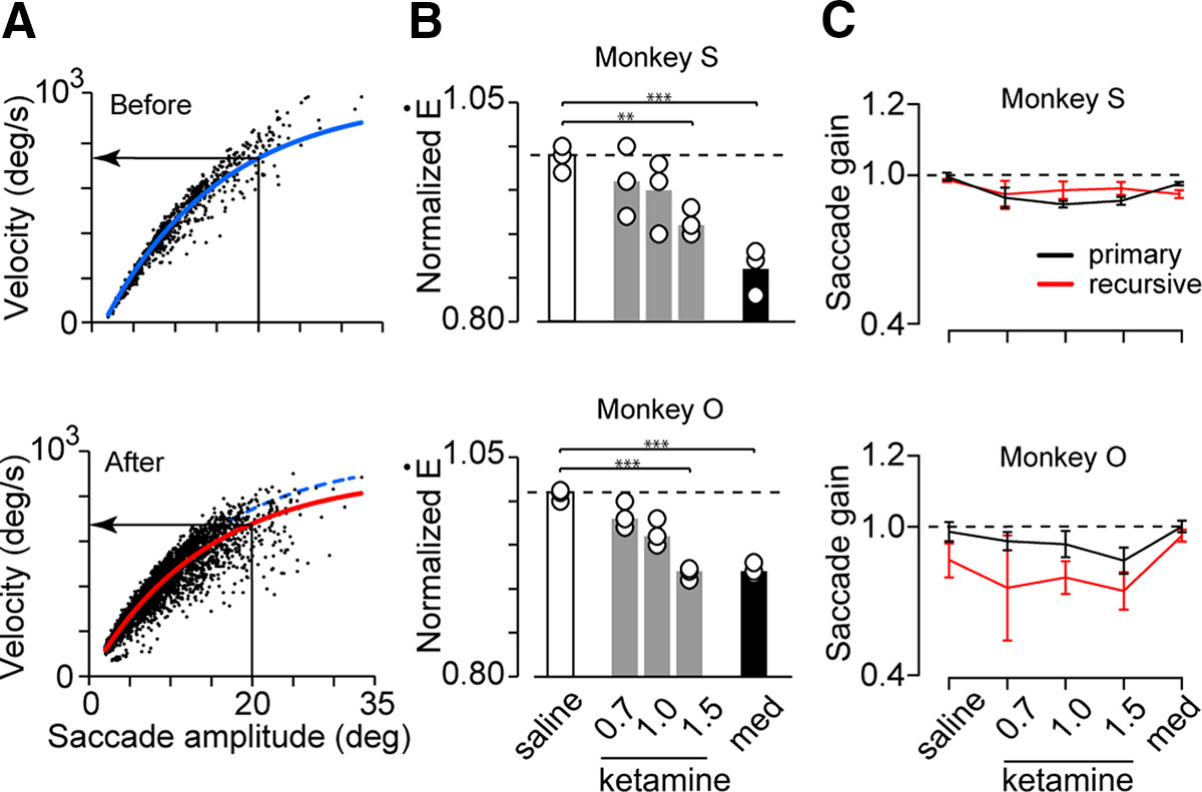
Effects of ketamine administration on saccade velocity and amplitude. ***A***, Relationships between saccade amplitude and peak velocity (main sequence) before (top panel) and 60 min following ketamine administration (1.5 mg/kg, bottom) in monkey S. Blue and red solid lines show the best-fit exponential curves (least squares) for the preinjection and postinjection data, respectively. The blue dashed curve in the bottom panel duplicates the one in the top panel for comparison. The leftward arrow indicates the estimated peak velocity for the 20° saccade. ***B***, Peak velocity following intramuscular injection of saline or drugs (0.7, 1.0, and 1.5 mg/kg ketamine and 0.01 mg/kg medetomidine) normalized for the preinjection data. The circles indicate individual experiments and bars denote the means of three sessions; **p <* *0.01, ***p <* *10^−3^ for multiple comparisons (Dunnett’s tests compared with saline). ***C***, Gains of primary (black) and recursive (red) saccades. Error bar indicates 1 SD for sessions.

**Figure 5.**
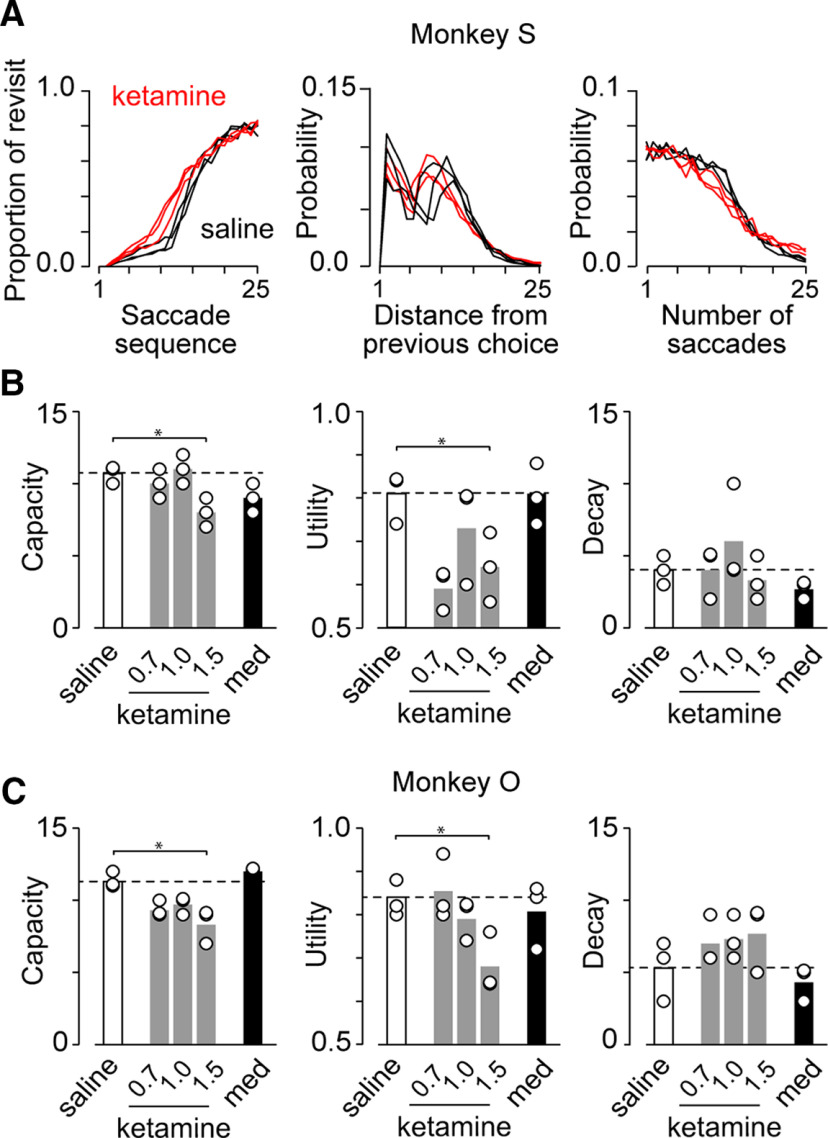
Effects of ketamine administration on model parameters. ***A***, Comparison of the best-fit data following saline (black traces) and ketamine (1.5 mg/kg, red traces) injection in monkey S. ***B***, ***C***, Three parameters were derived from the foraging model for different drug conditions. Data were collected 60 min after injection. The conventions are the same as those in Figure 4*B*.

**Figure 6.**
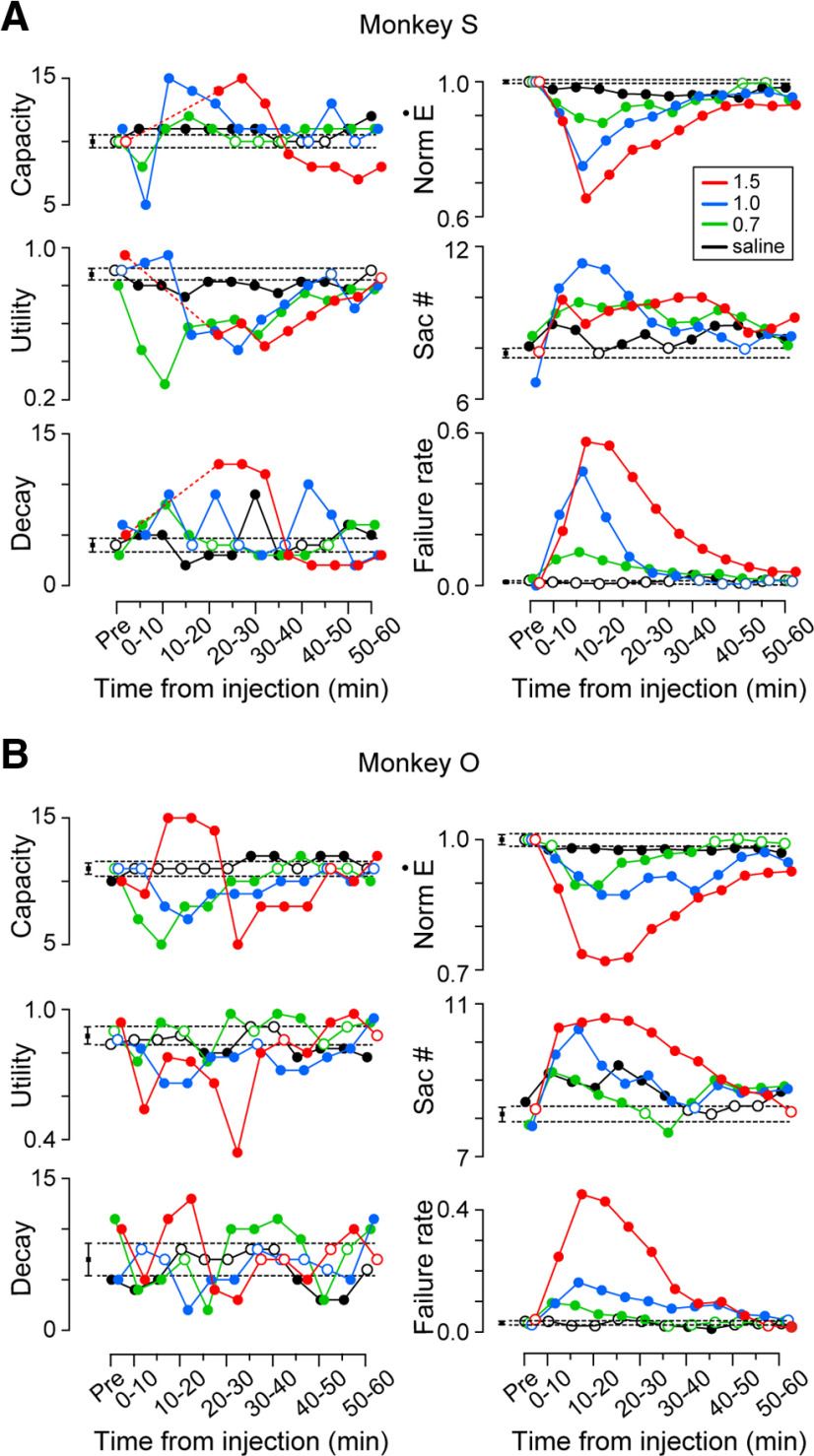
Time courses of foraging model parameters (left column) and eye movement parameters (right) following ketamine administration in monkeys S (***A***) and O (***B***). Data points connected with lines indicate single experimental conditions (data from three sessions). Different colors indicate different drug conditions. The left side black square indicates the preinjection data for all conditions (12 sessions). The error bars and horizontal dashed lines indicate the 95% confidence interval of the bootstrap data. Filled circles denote statistically significant modulations compared with the preinjection data. Data points for different drug conditions are slightly jittered horizontally for presentation purposes only.

We conducted two series of experiments: behavioral and pharmacological. In the behavioral experiments, all three monkeys performed the oculomotor foraging task for >60 min per session (nine, nine, and four sessions for monkeys S, O, and E, respectively; Fig. 2). We obtained less data from monkey E because she was also assigned to other projects. In the pharmacological experiments, only two monkeys (S and O) were used (Figs. 3-6). In each daily session, the animals received a single intramuscular injection of either normal saline (control), ketamine (0.7, 1.0, and 1.5 mg/kg), or medetomidine (α2 adrenoceptor agonist, 0.01 mg/kg) into the right femoral quadriceps after a block of 100 baseline (preinjection) trials. Monkeys continued to perform the behavioral task >60 min after the injection. The dosage of ketamine was determined based on previous pharmacological studies in monkeys (Taffe et al., 2002; Stoet and Snyder, 2006; Pouget et al., 2010; Ma et al., 2018) and humans (Ghoneim et al., 1985; Morgan et al., 2004), so that the animals could actively perform the task following injection. For the medetomidine experiments, ∼10% of the anesthetic dose was used. The injection volume was 1.0 ml for all experiments. Pharmacological experiments were separated by at least 3 d to avoid cumulative dosing effects. Different drug and dosage conditions were randomly ordered.

### Data acquisition and analysis

Eye position signals were digitized at 16-bit resolution and sampled at 1 kHz along with the event timestamps. Data were saved in files during the experiments for the subsequent offline analyses, which were performed using MATLAB (MathWorks). Saccades were detected when angular eye velocity exceeded 70°/s, eye acceleration exceeded 1000°/s^2^, and eye displacement was >2°. Trials were excluded from the analysis if they looked at the area outside of the virtual window (33° × 48°: the area of possible stimulus locations plus 4° margin) because of lack of concentration on the task (1.2%, 0.94%, and 7.90% in monkeys S, O, and E, respectively). Animals tended to look neighboring objects in initial sequences of each trial. To confirm this, we compared the means of initial five saccade amplitudes with those of the distances of five pairs of targets randomly chosen (100 iterations) in each monkey. When calculating the gain for each saccade, the saccade vector was first projected in the direction of the target, then the component saccade amplitude was divided by the target distance from the position of fixation (Fig. 4*C*).

It is well known that subanesthetic dose of ketamine can cause changes in oculomotor performance such as postsaccadic ocular drift and prolonged reaction time (Godaux et al., 1990; Mettens et al., 1990; Shen et al., 2010). To quantify these effects, we measured eye displacement during 100 ms from saccade termination (drift size) and every fixation interval from saccade end to the following saccade onset (intersaccadic interval; ISI). For each pharmacological experiment, we also examined eye velocity to confirm the drug effects. For the relationship between saccade amplitude and peak velocity (main sequence), we fitted an exponential curve of the following formula using the least squares method:

$$Ė={Ė}_{\text{max}}\text{ * [1 - exp(τ * Amp)]},$$

where Ė and Amp indicated peak angular velocity and amplitude of individual saccades, respectively, and Ė_max_ denoted the maximal value of Ė for each experiment (Fig. 4*A*). We estimated the peak velocity of the 20° saccade from the fitted curve, and the data obtained after drug (or saline) administration were normalized for the preinjection data (Fig. 4*B*,*C*).

In our foraging task, the optimal strategy to obtain rewards as early as possible was to remember the visited object and select a new object for each saccade. We assume that the rate of revisiting movements to the same object may depend on the amount of memory capacity and utility. For each experimental session, we computed the proportion of recursive behavior for every saccade sequence, the distribution of the distance from the previous same choice (i.e., the number of intervening saccades between the choices), and the distribution of saccade number in each trial (Fig. 2*A*). These data were normalized and were compared with the data generated by the foraging model, which incorporated parameters for WM and utility (Fig. 1*B*; see the following section).

### Foraging model

The foraging model was modified from the previous models of visual search (Horowitz, 2006). The model was defined by three parameters: (1) memory capacity, (2) memory decay, and (3) utility rate. As in the previous models, the memory capacity defined the maximal number of items stored in WM and ranged from 1 to 15. The memory decay was equivalent to the decay rate in the previous model (Horowitz, 2006) and defined the order of memory loss when the memory storage was saturated. For a value of unity, the oldest memory was replaced with the memory of the new item during visual search. For a value of 2, either of the oldest two memories was removed at equal probability. For a value equal to the memory capacity, the subjects randomly forgot one item regardless of their registered order. The utility rate was a newly developed parameter in our foraging model. The previous models of visual search incorporated the recall probability, which defined the probability of remembering all visited items (Arani et al., 1984; Horowitz, 2006). Similarly, the utility rate in our model defined the probability of referring to short-term memory to avoid recursive choices. When a value is unity, the subjects only choose from the items that are not registered in WM (that is, perfect use of WM). When a value is zero, the subjects randomly explore the object for every saccade.

We performed a Monte Carlo simulation (5000 iterations) for each set of three parameters to predict the agent’s behavior. The memory capacity and memory decay varied between 1 and 15, and the utility rate varied from 0.0 to 1.0 in steps of 0.02. Consequently, a total of 6120 sets of parameters were examined. For each set, we computed the three aforementioned distributions of revisiting behavior (Fig. 1*C*). We searched for the set of parameters that best accounted for the actual behavioral data (Fig. 2*A*). To do this, the coefficient of determination (CD) was calculated for each set of parameters using the following formula (Gomi et al., 1998):

$${\text{CD}}_{\text{i}}=\text{ 1 }-{\left(\text{M }-{\text{ S}}_{\text{i}}\right)}^{\text{2}}/\sum_{}^{}{\left[\text{M }-\text{ mean}\left({\text{S}}_{\text{i}}\right)\right]}^{\text{2}},$$

where S_i_ is the three distributions produced by the simulation for the i-th set of parameters and M is the actual data obtained from monkeys. Both S_i_ and M were normalized so that the sum of each of the three distributions had a value of unity. The CD approached 1.0 when the foraging model was able to explain the actual data. We searched for the optimal parameters from the 6120 combinations that maximized CD for each animal and experiment.

When we assessed the time courses of model parameters following ketamine administration (Fig. 6), we initially combined the data from three sessions in each drug condition and then divided the data for every 10-min window (5-min steps) following the injection. For each time point, we searched for the optimal set of model parameters using the combined data from three sessions. For the preinjection control, we also combined the data of all 12 sessions (saline and three ketamine conditions) for each monkey and searched for the optimal model parameters (Fig. 6, black squares). The range of significant values for each parameter was computed by resampling the combined preinjection data (1000 iterations). Any parameter following drug injection was statistically significant if it deviated from the middle 95% of the bootstrap data (Fig. 6, filled circles).

### Code availability

The simulation of the foraging model was performed using MATLAB running on Windows 10 (Dell XPS 8930) or macOS Catalina (MacBook Pro). The code for the simulation is provided upon reasonable request to the authors.

## Results

### Estimation of behavioral parameters using the foraging model

In the oculomotor foraging task, three monkeys searched for a target among 15 identical objects by generating sequential saccades within 6 s (Fig. 1*A*). The stimulus locations were randomly chosen from 76 candidate locations in each trial. The optimal strategy for the animals to obtain early rewards was to minimize revisiting the same objects. Our monkeys tended to select from neighboring items, although obvious strategies, such as looking from the end in order, were never observed. In all animals, the amplitudes of the initial five saccades in the sequence were statistically smaller than those expected from random choice (9.16 ± 1.02 vs 19.32 ± 0.15, paired *t* test, *t*_(21)_ = 48.95, *p *=* *10^−23^). Overall, the animals performed the task very well and successfully reached the reward-associated target in most of the trials (90.8%, 97.6%, and 87.2% in monkeys S, O, and E, respectively).

We devised a model to explain the animals’ behavior during the task (Fig. 1*B*). The foraging model incorporated three parameters: (1) memory capacity, (2) memory decay, and (3) utility rate (see Materials and Methods). Briefly, memory capacity limited the maximal number of objects stored in short-term memory. Memory decay defined the order of memory loss when the storage was saturated. Memory utility rate determined the occurrence of exploitative behavior that regularly selected a new object with the use of WM. We evaluated the agent’s performance by plotting three distributions of revisiting behavior derived from Monte Carlo simulations (Fig. 1*C*). The proportion of revisiting behavior for each saccade sequence was sensitive to both memory capacity and utility rate but not to memory decay (Fig. 1*C*, left column). When the number of intervening saccades from the previous choice for each revisiting behavior was considered, its distribution was significantly altered for changes in any of the three parameters (Fig. 1*C*, middle column). The distribution of saccade number in each trial was again sensitive to both memory capacity and utility rate, whereas the changes in memory decay were ineffective (Fig. 1*C*, right column).

We attempted to account for animals’ behavior using the foraging model. Figure 2*A* shows a plot of the data obtained from multiple sessions in three monkeys (gray histograms) with the best-fit distributions generated by the foraging model (color traces). The optimal parameters and the CD computed for the entire distribution are shown on the right panel of Figure 2*A* for each monkey. Although the optimal parameters varied between animals, the CD was always >0.92. We also computed the optimal parameters for individual sessions (Fig. 2*B*). Although the model fit became somewhat worse for the smaller number of trials in each session (ranging from 358 to 1173 trials), the CD for the optimal parameters averaged 0.90 ± 0.05 (SD, *n *=* *22) and was always >0.74. We found that there was some daily variability of optimal parameters in each monkey, but the variation between sessions appeared to be less than that between animals. To quantify this, we computed the squared sum of the normalized difference in parameters between every pair of sessions within and across monkeys. The values were significantly smaller for the variation between sessions than for the variation across monkeys (two-tailed *t* test, *t*_(229)_ = −4.76, *p *=* *10^−6^), indicating that the model parameters were mostly unique to each animal despite the small daily variations.

Although the memory capacity and utility rate reflected distinct neural mechanisms (Fig. 1*B*), the expected changes in agent’s performance because of changes in these parameters were qualitatively similar (Fig. 1*C*, top two rows). This suggested that the estimate of these two parameters from behavioral data might be inversely correlated. However, we found that these values across 22 sessions were not significantly correlated (*r *=* *0.15, *p *=* *0.50). Combined with the fact that recursive choice in very early saccade sequences can only be accounted for by the utility rate, we conclude that the three parameters of the model can be used for quantitative measures of different functions.

### Changes in eye movement parameters following ketamine administration

We next examined the changes in behavioral parameters after administration of low-dose ketamine (an NMDA receptor antagonist). Although subanesthetic dose of ketamine is known to alter a variety of cognitive functions (Krystal et al., 1994; Berman et al., 2000; Condy et al., 2005), it can also change oculomotor parameters, causing postsaccadic ocular drift and prolonged saccade latency (Godaux et al., 1990; Mettens et al., 1990; Shen et al., 2010). Therefore, we firstly examined eye movement parameters during the oculomotor foraging task after ketamine administration.

We found that our monkeys also showed postsaccadic drift and prolonged fixation interval shortly after ketamine injection (Fig. 3*A*). To examine the time course of the drug effects, we measured the drift size, ISI, and saccade number during every 10 min (in 5-min steps) following injection (Fig. 3*B*). In both monkeys, ketamine clearly increased the drift size and ISI in a dose-dependent manner. These changes reached the maximum within 15 min following injection and rapidly returned to the normal level. In addition, ketamine dramatically (monkey S) or slightly (monkey O) decreased the number of saccades during the foraging task. Administration of a subanesthetic dosage of medetomidine (α_2_ agonist) did not alter postsaccadic stability or ISI but decreased saccade frequency after 30 min of injection. Despite the postsaccadic slow drift after ketamine administration, we were able to reliably detect individual targeting saccades using the combination of velocity, acceleration, and amplitude criteria (see Materials and Methods).

Although monkeys were well-motivated to perform the task following ketamine or medetomidine injection, we found that their eye velocity decreased slightly throughout the experiment. Figure 4*A* shows the relationships between saccade amplitude and peak angular eye velocity before (top panel) and after ketamine injection (bottom). For quantification, the data were fitted with an exponential curve (least squares), and eye velocity for the 20° saccade was estimated. Figure 4*B* summarizes the eye velocity following saline or drug injections normalized for the preinjection data. In both monkeys, eye velocity significantly altered after drug injections (Dunnett’s test, saline vs 1.5 mg/kg ketamine, *p *<* *0.01 and 10^−4^ for monkeys S and O, respectively; saline vs medetomidine, *p *<* *10^−3^ and 10^−4^). Furthermore, the effect of ketamine was dose-dependent in one monkey (Spearman’s rank correlation coefficient, *r*_s(9)_ = −0.53, *p *=* *0.16 and *r*_s(9)_ = −0.87, *p *<* *0.01 for monkeys S and O, respectively). In both monkeys, medetomidine reduced eye velocity by as much as or greater than that after 1.5 mg/kg ketamine administration (Fig. 4*B*).

The drug effects on eye movements were further examined separately for primary and recursive saccades during the task. We firstly computed the gain for each saccade (ratio of saccade amplitude divided by target eccentricity; see Materials and Methods) and compared them across drug conditions (Fig. 4*C*). In one monkey (S), saccade gain tended to be larger for recursive than primary saccades and ketamine dosage slightly but significantly affected (two-way ANOVA, saccade type: *F*_(1,20)_ = 6.9, *p < *0.05, drug: *F*_(4,20)_ = 31.0, *p < *10^−8^, interaction: *F*_(4,20)_ = 13.6, *p < *10^−5^). In the other monkey (O), saccade gain was smaller for recursive than primary saccades (saccade type: *F*_(1,20)_ = 58.4, *p < *10^−7^, drug: *F*_(4,9)_ = 16.3, *p <*10^−6^, interaction: *F*_(4,20)_ = 2.46, *p *=* *0.08), even following saline injection. However, when peak velocity of 20° saccade was estimated, each drug similarly reduced the values for the primary and recursive saccades (1.5 mg/kg ketamine: 7.8% and 8.1% reduction of primary saccades, 10.2% and 11.0% reduction of recursive saccades for monkeys S and O, respectively; medetomidine: 6.2% and 6.5% reduction of primary saccades, 15.7% and 9.4% reduction of recursive saccades).

### Changes in WM performance following ketamine administration

We next compared the parameters of the fitted foraging model for the data following saline injection with those following drug injections. Figure 5*A* displays the data derived from the best-fit models for the three ketamine (1.5 mg/kg, red lines) and three saline (black lines) experiments in monkey S. Despite slight intersession variability, a clear difference between saline and ketamine conditions can be seen. Figure 5*B*,*C* summarizes the parameters for the best-fit model in two monkeys. In all cases, CD was >0.84 and averaged 0.92 ± 0.03 (SD, *n *=* *30). For both animals, the memory capacity was significantly altered following ketamine injection (Dunnett’s test, saline vs 1.5 mg/kg ketamine, *p *<* *0.05 for both monkeys) but not following medetomidine injection (*p *=* *0.22 and 0.89 for monkeys S and O, respectively; Fig. 5*B*,*C*, left panels). The ketamine effect on memory capacity was not dose-dependent (Spearman’s rank correlation coefficient, *r*_s(9)_ = −0.59, *p *=* *0.12 and *r*_s(9)_ = 0.06, *p *=* *0.89 for monkeys S and O, respectively). Similarly, the utility rate was significantly reduced following ketamine injection but not following medetomidine injection (Dunnett’s test, saline vs 1.5 mg/kg ketamine, *p *<* *0.05 for both monkeys; saline vs medetomidine, *p *=* *0.91 and *p *=* *0.82; Fig. 5*B*,*C*, middle panels). A significant dose effect was found in one monkey (*r*_s(9)_ = −0.79, *p *<* *0.05 for monkey O, *r*_s(9)_ = 0.24, *p *=* *0.55 for monkey S). By contrast, we failed to find any significant difference in memory decay following either drug injection (saline vs 1.5 mg/kg ketamine, *p *=* *0.73 and *p *=* *1.00 for monkeys S and O, respectively; saline vs medetomidine, *p *=* *0.53 and *p *=* *0.69; Fig. 5*B*,*C*, right panels). On average, ketamine administration caused 9.4% and 19.6% reductions in memory capacity, 78.2% and 34.7% reductions in utility rate, and 11.1% and 37.5% increases in memory decay for monkeys S and O, respectively.

We obtained similar results when the first 15 min of data following ketamine injection were excluded from the analysis to rule out the possible confounding effects of the oculomotor abnormalities shown in Figure 3. The memory capacity and utility rate significantly decreased following ketamine injection (Dunnett’s test, memory capacity: saline vs 1.5 mg/kg ketamine, *p *<* *0.05 and *p *<* *0.05 for monkeys S and O, respectively, saline vs medetomidine, *p *=* *0.22 and *p *=* *0.89; utility rate: saline vs 1.5 mg/kg ketamine, *p *<* *0.05 and *p *<* *0.05, saline vs medetomidine, *p *=* *0.85 and *p *=* *0.77; memory decay: saline vs 1.5 mg/kg ketamine, *p *=* *0.73 and *p *=* *0.74, saline vs medetomidine, *p *=* *0.53 and *p *=* *0.65).

Figure 6 illustrates the time courses of the parameters of the optimal model and saccades following ketamine or saline injection. Each circle connected with lines represents the data for three combined sessions, while the black square on the left side of each panel represents the preinjection data of 12 sessions. Because monkey S greatly decreased the number of saccades shortly after 1.5 mg/kg ketamine injection (Fig. 3*B*, bottom panel), we were unable to reliably estimate the model parameters (CD < 0.7) and therefore removed these data from the plots (Fig. 6*A*, left panels, three data points replaced with dashed lines). For all the data plotted in Figure 6*A*,*B*, CD for the model fit was >0.79 and averaged 0.88 ± 0.04 (SD, *n *=* *85). In both monkeys, memory capacity and memory decay changed in various directions following ketamine injection and deviated from the 95% confidence intervals of the data obtained before the injection (as indicated by error bars of black squares and horizontal dashed lines). Conversely, the utility rate decreased consistently over time in a dose-dependent manner. Normalized eye velocity for 20° saccade (Ė) was also reduced consistently following ketamine administration, and the average number of saccades in each trial and the rate of failed trials increased in a dose-dependent manner. For both monkeys, the utility rate and saccade parameters after ketamine administration were correlated significantly (Spearman’s rank correlation coefficient, eye velocity: *r*_s(33)_ = 0.58, *p *<* *10^−3^ and *r*_s(36)_ = 0.55, *p *<* *10^−3^ for monkeys S and O, respectively; saccade number: *r*_s(33)_ = −0.49, *p *<* *0.01 and *r*_s(36)_ = −0.57, *p *<* *10^−3^; failure rate: *r*_s(33)_ = −0.52, *p *<* *0.01 and *r*_s(36)_ = −0.76, *p <*10^−7^). In contrast, memory capacity and memory decay only partially correlated with saccade parameters (capacity vs velocity: *r*_s(33)_ = −0.37, *p *<* *0.05 and *r*_s(36)_ = 0.28, *p *=* *0.10 for monkeys S and O, respectively; capacity vs saccade number: *r*_s(33)_ = 0.25, *p *=* *0.17 and *r*_s(36)_ = −0.15, *p *=* *0.37; capacity vs failure rate: *r*_s(33)_ = 0.17, *p *=* *0.34 and *r*_s(36)_ = −0.32, *p *=* *0.06; decay vs velocity: *r*_s(33)_ = −0.29, *p *=* *0.10 and *r*_s(36)_ = 0.20, *p* =0.25; decay vs saccade number: *r*_s(33)_ = 0.24, *p *=* *0.18 and *r*_s(36)_ = −0.25, *p* = 0.14; decay vs failure rate: *r*_s(33)_ = 0.23, *p *=* *0.20 and *r*_s(36)_ = −0.33, *p *<* *0.05). Thus, among the three model parameters, a significant correlation with the changes in saccade parameters following ketamine administration was only found for the utility rate. We also found that memory capacity, utility rate, and memory decay were not significantly correlated with behavioral parameters in medetomidine experiments (Spearman’s correlation coefficient ranged from −0.55 to 0.51, *p *>* *0.05) with a few exceptions (utility rate vs velocity for monkey S, *r*_s(12)_ = −0.72, *p *<* *0.01; utility rate vs failure rate for monkey S, *r*_s(12)_ = −0.58, *p *<* *0.05; memory decay vs velocity for monkey S, *r*_s(12)_ = 0.62, *p *<* *0.05; memory capacity vs saccade number for monkey O, *r*_s(12)_ = 0.86, *p *<* *10^−3^).

Finally, we obtained similar results when performing correlation analysis after excluding the first 15 min of data following injection. Among the three model parameters, significant correlations with changes in saccade parameters were consistently found for utility rate and also for some pairs with memory capacity (utility rate vs velocity: *r*_s(33)_ = 0.85, *p *<* *10^−8^ and *r*_s(34)_ = 0.61, *p *<* *10^−3^ for monkeys S and O, respectively; utility rate vs saccade number: *r*_s(33)_ = −0.77, *p *<* *10^−6^ and *r*_s(34)_ = −0.49, *p *<* *0.01; utility rate vs failure rate: *r*_s(33)_ = −0.78, *p *<* *10^−6^ and *r*_s(34)_ = −0.74, *p <*10^−5^; capacity vs velocity: *r*_s(33)_ = −0.35, *p *=* *0.07 and *r*_s(34)_ = 0.41, *p *=* *0.03; capacity vs saccade number: *r*_s(33)_ = 0.29, *p *=* *0.12 and *r*_s(34)_ = −0.19, *p *=* *0.33; capacity vs failure rate: *r*_s(33)_ = 0.20, *p *=* *0.31 and *r*_s(34)_ = −0.42, *p *=* *0.02; decay vs velocity: *r*_s(33)_ = −0.17, *p *=* *0.39 and *r*_s(34)_ = 0.29, *p *=* *0.12; decay vs saccade number: *r*_s(33)_ = 0.11, *p *=* *0.57 and *r*_s(34)_ = −0.32, *p *=* *0.08; decay vs failure rate, *r*_s(33)_ = 0.04, *p *=* *0.85 and *r*_s(34)_ = −0.42, *p *<* *0.05).

## Discussion

In the present study, monkeys were trained to sequentially search for a target among visually identical objects to receive a liquid reward. The task of searching for stimuli for reward was previously called the “non-visual search task” (Collin et al., 1982; Passingham, 1985) or the “scanning task” (Sommer, 1994), but here, we named it “oculomotor foraging task” after the “visual foraging task” developed by Mirpour et al. (2009, 2019). The animals performed the task very well (>87% correct rate) and seemed to use the optimal strategy to obtain an early reward by minimizing recursive saccades. According to our foraging model, the memory capacity of the three monkeys ranged from 7 to 10, and the utility rate ranged from 0.74 to 0.90. We also found that systemic administration of low doses of ketamine reduced the memory capacity and utility rate by 15% and 57%, respectively. These results indicated that low doses of ketamine disrupted the WM performance, largely by promoting exploratory behavior during the foraging task.

### Validity and usefulness of the foraging model

In our experiments, the behavioral parameters derived from the optimal foraging model varied somewhat from session to session, but the variance between sessions was significantly less than that between animals (Fig. 2*B*). These results suggest that our foraging model rigorously evaluated WM performance in individual monkeys. Furthermore, while the changes in utility rate and memory capacity similarly altered the agent’s performance (Fig. 1*C*), these two parameters did not correlate across sessions, indicating that these parameters separately reflected different brain functions. In a previous study, Mirpour et al. (2009) examined the oculomotor performance of monkeys during the visual foraging task, where five potential targets and five distractors were simultaneously presented. The animals’ behavior was well explained by their model incorporating two parameters: target identification probability and recall probability. The former was the probability that the observer distinguished targets from distractors, and the latter was the probability that the observer perfectly remembered the previously visited items. The monkeys’ performance suggested that they were always able to discriminate targets from distractors (target identification probability = 1.0) and remembered most of the previously visited objects (recall probability > 0.92). This excellent memory performance was likely because of the relatively small number of potential targets, which was limited to five. Conversely, in our foraging paradigm, the number of potential targets was 15, and no explicit distractor was presented so that the memory load was much greater than in the previous study. Thus, our behavioral task and proposed model were distinct from the previous ones and uniquely examined WM performance.

Although the estimated memory capacity ranged from 7 to 10 items in our monkeys (Fig. 2*A*), previous studies using the change detection paradigms estimated the memory capacity to be four or less in both monkeys (Buschman et al., 2011; Elmore et al., 2011; Heyselaar et al., 2011) and humans (Luck and Vogel, 1997; Saiki, 2002). In our foraging task, the animals could use spatial cue, such as geometric distribution and object clustering, to remember many visited objects. Indeed, while obvious strategies, such as looking from the end in order, were not observed, our monkeys tended to choose from neighboring items. Furthermore, although the change detection paradigm requires memory for the location and identity of each object, the visited objects in visual search can be simply labeled as non-targets and their identities are task-irrelevant. A previous study of visual search in humans estimated the memory capacity to be >12 items, but the spatial resolution of memory was relatively low (Dickinson and Zelinsky, 2007). Thus, a more precise estimate of memory capacity might be possible by incorporating factors of relative object locations into the foraging model.

Our foraging model incorporated two additional parameters along with memory capacity. Memory utility rate indicated the recall probability of short-term memory; otherwise, the subjects randomly searched for the target. This was representative of a natural situation in which one cannot remember the items and impulsively selects something unrelated. Memory decay indicated the order of memory loss when the WM storage was saturated. The value was small if the retention of short-term memory was stable, and it was large if the memory of the visited items was randomly lost. In our experiments, the memory decay varied somewhat between monkeys and ranged from 3 to 10 (Fig. 2). Although the effect of memory decay on behavioral distribution was less than the effects of the other two parameters (Fig. 1*C*), it was helpful in accurately reproducing the behavioral distribution for trials with many recursive saccades. All three parameters were mostly stable in each animal and might reflect different aspects of WM function.

Most clinically available WM scales only consider reaction time and correct rate, and are sometimes difficult to apply to patients and small children because the testing procedure is slightly complicated and time-consuming. Contrary to these limitations, our oculomotor foraging task is based on a simple visual search without any difficult instructions (e.g., finding a target as quickly as possible) and can be used to evaluate several behavioral parameters as mentioned above. In recent years, inexpensive eye movement measuring devices have become available, so this behavioral task might be used as a simple clinical test. To this end, the application of the foraging task to various types of neurologic and psychiatric disorders as well as the flexibility and usefulness of the foraging model need to be investigated in future studies.

### Effects of low-dose ketamine on WM performance

Administration of low-dose ketamine (an NMDA receptor antagonist) has been shown to successfully produce WM dysfunction in experimental animals such as rodents (Wesierska et al., 1990; Enomoto and Floresco, 2009; Rushforth et al., 2011) and primates (Condy et al., 2005; Skoblenick and Everling, 2012; Blackman et al., 2013; Gil-da-Costa et al., 2013; Wang et al., 2013). Human subjects administered with a subanesthetic dosage of ketamine have also participated in neuropsychological studies as putative models of psychosis (Lahti et al., 2001; Xu et al., 2015). In this study, we found that low doses of ketamine consistently reduced the utility rate and eye velocity and elevated the rate of failed trials. Furthermore, they also reduced the memory capacity in both monkeys. Overall, the WM dysfunction following ketamine administration appeared to be mostly due to difficulty in using short-term memory, while a decrease in short-term memory capacity also contributed. The decreased memory capacity found in this study might be well explained by the previous network model of WM that incorporated recurrent circuits mediated by NMDA receptors (Lisman et al., 1998; Wang, 2001). In contrast, an α_2_ adrenoceptor agonist (medetomidine, 0.01 mg/kg) reduced eye velocity to the maximal dose of ketamine (1.5 mg/kg) but failed to alter the utility rate or the memory capacity. We also found that low-dose ketamine induced oculomotor abnormalities such as postsaccadic ocular drift, prolonged fixation interval, and decreased saccade number during the task, which peaked within 15 min following injection (Fig. 3). However, the ketamine effects on model parameters were consistently observed even when the first 15 min of data were excluded from the analysis. These results suggested that the changes in model parameters following ketamine administration were not merely due to changes in eye movement performance.

Examination of the time course of model parameters following ketamine injection revealed that memory capacity and memory decay were only transiently altered, and the direction of these changes did not match between monkeys (Fig. 6). Conversely, the utility rate consistently decreased over time in a dose-dependent manner. Furthermore, the utility rate alone correlated significantly with the rate of failed trials and eye velocity. These observations suggested that the changes in behavioral performance following ketamine administration might largely depend on the changes in memory utility. As short-term memory became unavailable, animals tended to make exploratory choices and increase recursive behaviors. Previous studies have reported that attentional deficits caused by PFC dysfunction may lead to reduced exploitative behavior (Barceló et al., 2000; Voytek et al., 2010). In relation to this, low doses of ketamine have been shown to increase perseverative errors in healthy individuals during the Wisconsin Card Sorting Test (Krystal et al., 2000). These previous findings are consistent with the notion that low doses of ketamine may interfere with the central executive function of WM rather than simply reducing the memory capacity.

Although significant changes in memory utility were found following ketamine (but not medetomidine) injection, it is highly conceivable that the effects on model parameters in our experimental conditions were not limited to those mediated by NMDA receptors. For example, the cholinergic system might affect memory utility by modulating the attentional state (Voytko et al., 1994; Disney et al., 2007; Thiele and Bellgrove, 2018; Sarter and Lustig, 2019) and the dopaminergic system may directly regulate the central executive functions (Barnes et al., 2011) and quality of short-term memory (Aigner et al., 1991; Castner et al., 2000; Wang et al., 2019). Thus, the effects of other pharmacological agents should be examined in future studies. Psychotropic agents that alter the model parameters in a more favorable direction could be used as potential treatment options for WM dysfunctions. In addition, our experimental paradigm and foraging model with minor modifications may be applicable to humans to assess WM performance in a clinical setting.

## Synthesis

Reviewing Editor: Nicholas J. Priebe, University of Texas at Austin Decisions are customarily a result of the Reviewing Editor and the peer reviewers coming together and discussing their recommendations until a consensus is reached. When revisions are invited, a fact-based synthesis statement explaining their decision and outlining what is needed to prepare a revision will be listed below. The following reviewer(s) agreed to reveal their identity: NONE.

SYNOPSIS

The revision of your manuscript was reviewed by both the reviewing editor and one reviewer. The revision addresses the concerns previously raised. However, it is necessary to include details about the code used for the foraging model. In the Materials and Methods section there should be a heading titled Code Accessibility that indicates how the code can be accessed, as well as the type of computer or operating system used to run the code.
